# Climatic Factors Influencing the Anthrax Outbreak of 2016 in Siberia, Russia

**DOI:** 10.1007/s10393-021-01549-5

**Published:** 2021-08-28

**Authors:** Ekaterina Ezhova, Dmitry Orlov, Elli Suhonen, Dmitry Kaverin, Alexander Mahura, Victor Gennadinik, Ilmo Kukkonen, Dmitry Drozdov, Hanna K. Lappalainen, Vladimir Melnikov, Tuukka Petäjä, Veli-Matti Kerminen, Sergey Zilitinkevich, Svetlana M. Malkhazova, Torben R. Christensen, Markku Kulmala

**Affiliations:** 1 Institute for Atmospheric and Earth System Research (INAR)/Physics, Faculty of Science, University of Helsinki, Helsinki, Finland; 2 Department of Biogeography, Faculty of Geography, Lomonosov Moscow State University, Moscow, Russia; 3 Institute of Biology of Komi Scientific Center of the Russian Academy of Sciences, Syktyvkar, Russia; 4 International Centre of Cryology and Cryosophy, University of Tyumen, Tyumen, Russia; 5 Department of Geosciences and Geography, Faculty of Science, University of Helsinki, Helsinki, Finland; 6 Earth Cryosphere Institute, Siberian Branch of the Russian Academy of Sciences, Tyumen, Russia; 7 Hydrogeological Department, Faculty of Engineering Geology, Russian State Geological Prospecting University, Moscow, Russia; 8 Department of Earth Cryology, Industrial University of Tyumen’, Tyumen, Russia; 9 Tyumen Scientific Center of Siberian Branch of the Russian Academy of Sciences, Tyumen, Russia; 10 Finnish Meteorological Institute, Helsinki, Finland; 11 Department of Bioscience, Arctic Research Centre, Aarhus University, Aarhus, Denmark

**Keywords:** Anthrax, Permafrost, Climate, Snow, Drought, Siberia

## Abstract

In 2016, an outbreak of anthrax killing thousands of reindeer and affecting dozens of humans occurred on the Yamal peninsula, Northwest Siberia, after 70 years of epidemiological situation without outbreaks. The trigger of the outbreak has been ascribed to the activation of spores due to permafrost thaw that was accelerated during the summer heat wave. The focus of our study is on the dynamics of local environmental factors in connection with the observed anthrax revival. We show that permafrost was thawing rapidly for already 6 years before the outbreak. During 2011–2016, relatively warm years were followed by cold years with a thick snow cover, preventing freezing of the soil. Furthermore, the spread of anthrax was likely intensified by an extremely dry summer of 2016. Concurrent with the long-term decreasing trend in the regional annual precipitation, the rainfall in July 2016 was less than 10% of its 30-year mean value. We conclude that epidemiological situation of anthrax in the previously contaminated Arctic regions requires monitoring of climatic factors such as warming and precipitation extremes.

**Supplementary Information:**

The online version contains supplementary material available at 10.1007/s10393-021-01549-5.

## Introduction

Anthrax has been known since ancient times with the first descriptions dating back to Hippocrates, fifth century BC (Schwartz 2009), and it is endemic to all the continents except Antarctica (Dragon and Rennie 1995; WHO 2008; Malkhazova et al. 2019; Carlson et al. 2019). The disease is caused by the soil bacteria *Bacillus anthracis*. The bacteria are sensitive to the moisture, acidity and organic content of soils, and their life cycles are influenced by climatic factors, such as ambient temperature and precipitation (Dragon and Rennie 1995; WHO 2008; Waits et al. 2018; Walsh et al. 2018; Malkhazova et al. 2019; Carlson et al. 2019). Therefore, some regions are more affected by anthrax than others (for a compilation map based on outbreaks from 2005 to 2016, see Carlson et al. 2019). In the regions endemic for anthrax, high incidences occur during dry and warm periods following intensive precipitation, explaining localities of the major outbreaks in countries with warm climates such as Turkey, Ethiopia, South Africa etc. (Malkhazova et al. 2019; Carlson et al. 2019). In spite of that, anthrax can survive cold climates as well. The vast geographical range of anthrax and risk of recurrence after years or even decades (Dragon and Rennie 1995) is due to high resistivity of spores to unfavorable conditions and their ability to effectively reproduce themselves (Driks 2009).

In the beginning of the twentieth century, a northern part of Western Siberia was experiencing severe and recurring epizootics of anthrax: more than one million reindeer died (Popova et al. 2016). The affected territories include a large area on Yamal peninsula (Yamal district), somewhat smaller areas in the north of Nadym district and in the central Tazovsky district, and many small sites in Priuralsky, Nadym and Pur districts (Popova and Kulichenko 2017). Since the 1940s and up to 2007, vaccination of the reindeer population effectively eliminated the disease (Popova et al. 2016; Arkhangelskaya 2016). In 2007, a decade before the outbreak, the vaccination of the reindeer was halted (Popova et al. 2016; Arkhangelskaya 2016). During this decade, more than 200,000 samples of soil from 32 of known anthrax-contaminated areas of Yamal-Nenets Autonomous District were analyzed and none showed signs of *B. anthracis* (Shestakova 2016; Selyaninov et al. 2016). Vulnerability of bacteria to repetitive freeze–thaw cycles (Malkhazova et al. 2019) could lead to an eventual sanitation of the soil, given decades of epidemiological stability. Supporting the sanitation hypothesis, Cherkassky (2003) investigated 360 soil probes from contaminated areas on Yamal peninsula in 1968 and found that the soil pH was in the range of 3–5, i.e., below the threshold value of 6 (e.g., Van Ness 1971), and that the soil was poor in organics (humus content below 3%).

Although absent near the soil surface, the anthrax spores could remain intact in the carcasses of dead infected animals buried in permafrost. Its thawing due to a warming climate might revive previously frozen bacteria back to life (Popova et al. 2016; Goudarzi 2016; Coghlan 2016). A recent study found that *B. anthracis* strains isolated at Yamal were close to those isolated from permafrost in Yakutia (Timofeev et al. 2019), supporting the hypothesis about permafrost thawing as a trigger for the outbreak. Here, we considered regional (acting on the scales of ~ 100 km) weather and climate parameters, connected their recent dynamics to the dynamics of permafrost and addressed the existing hypotheses about the anthrax outbreak on Yamal. We started with active layer thickness (ALT) dynamics characterizing permafrost thawing in the sites near the outbreak location. Furthermore, we studied dynamics of the mean annual air temperature (MAAT), as well as snow depth and temperature-based indices, aiming to explain ALT dynamics. To account for the joint effect of warm and preceding cold season on ALT, we used frost numbers and found correlations between those and ALT. Finally, we outlined large-scale phenomena and processes potentially relevant for anthrax outbreaks in cold climates.

## Methods

**Figure 1:**
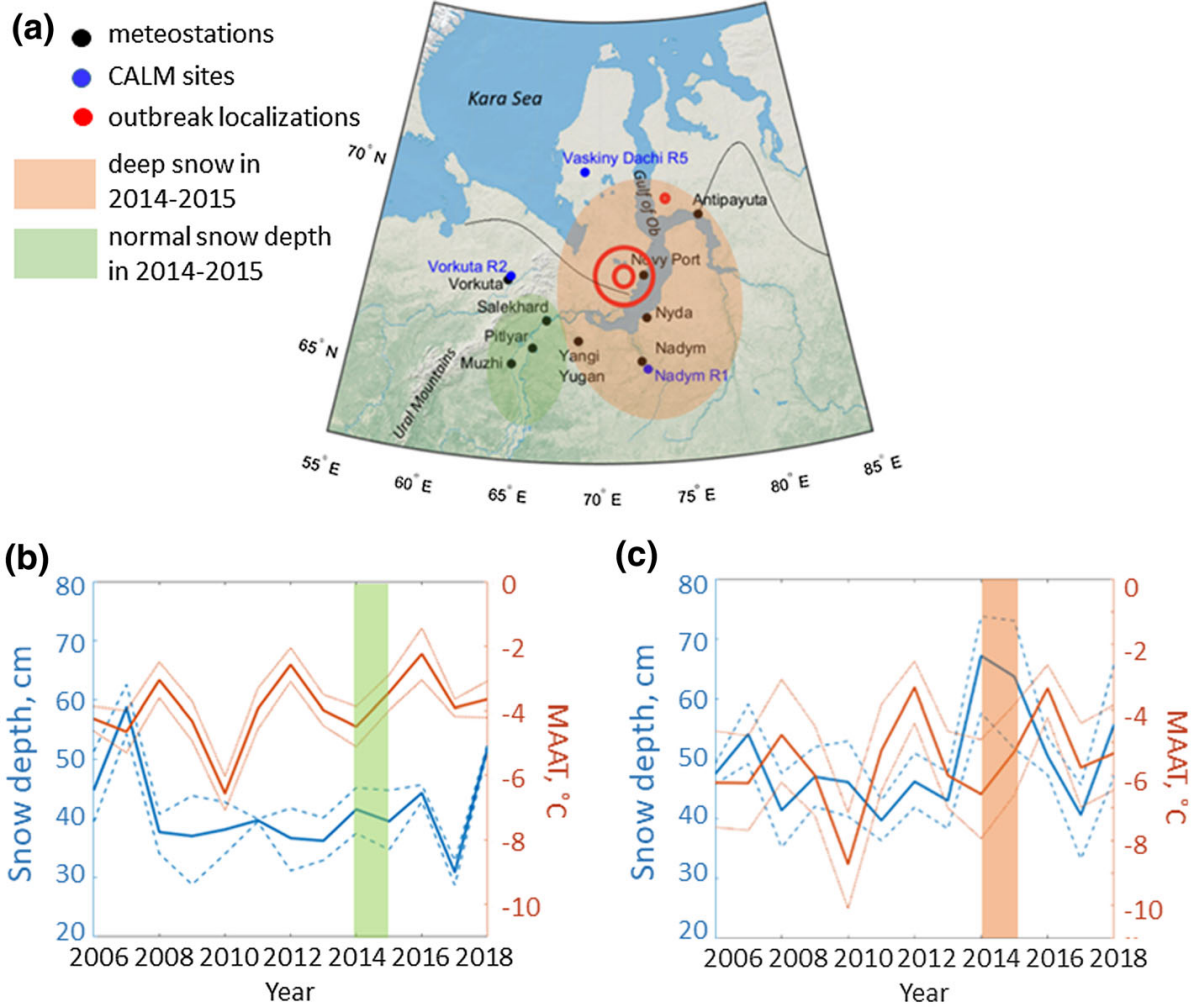
**a** Location of meteorological stations and Circumpolar Active Layer Monitoring (CALM) in Yamal-Nenets Autonomous District. Black curves separate continuous from discontinuous permafrost areas (continuous permafrost to the north). Red circles show areas of major anthrax epizootics, with 2650 reindeer and 36 human cases registered in Yamal district (near Novy Port) and 1 reindeer case—in Tazovsky district (near Antipayuta). **b** Median snow depth and mean annual air temperature at sites to the east of Ural mountains. Thin dashed and solid curves represent 25th and 75th quartiles. No increase in snow depth in 2014–2015. **c** Median snow depth and mean annual air temperature at the sites near the Gulf of Ob. Note an increase in snow depth in 2014–2015.

We used meteorological data sets and data on active layer thickness from several sites in Yamal-Nenets Autonomous District and Komi Republic, Russia, close to the location of the outbreak (Fig. 1; Popova et al. 2016). The medical geography data set giving some quantitative information on the outbreak can be found in Supplementary Information, Table S1. We started with the hypothesis that permafrost thawing could trigger an outbreak and first considered recent dynamics of permafrost and its link to climatic parameters. Then, we looked into summer precipitation, a parameter that could influence spreading of anthrax.

The active layer thickness, which is the maximum annual thaw depth, is the parameter characterizing thawing of permafrost. We estimated the effect of air temperature and snow on the soil surface temperature which determines the state and dynamics of permafrost. Temperature-based indices that prove useful in estimates of ALT are freezing and thawing indices (or degree-day sums) corresponding to the sums of daily mean temperatures during cold and warm seasons, respectively (absolute value in case of cold season, see subsection on freezing and thawing indices below). Cold and warm seasons are defined as the seasons when daily mean temperature is stably below and above 0°C, respectively. Air freezing and thawing indices can be derived from the air temperature, whereas surface freezing and thawing indices are calculated based on the surface temperature. The soil surface temperature is not routinely measured at meteorological stations, so we modelled the surface indices. From MAAT and snow depth, we estimated a freezing *n*-factor (e.g., Klene et al. 2001)—the ratio between the freezing indices for surface and air, which characterizes heat transfer between soil surface and air. In winter, the soil surface temperature is higher than the air temperature due to snow insulation, and the corresponding freezing indices are lower. Deep snow corresponds to *n*-factors close to zero, compared to no-snow conditions with *n*-factor equal to unity. We calculated the surface freezing index from the air freezing index and a freezing *n*-factor. The formula can be found in subsection ‘*n*-factors.’ After that, we used freezing and thawing indices for air and surface to construct air and surface frost numbers and studied their correlation with ALT.

### Active Layer Thickness

The ALT data used in this study were taken from the open database of Circumpolar Active Layer Monitoring network (CALM). Five measurement sites are located in Yamal-Nenets Autonomous District (Fig. 1, Table S2): one near Nadym (monitoring site R1, Bobrik et al. 2015), and four sites near Vaskiny Dachi (sites R5, R5A, R5B, R5C, Leibman et al. 2015). One site, Ayach-Yakha near Vorkuta (site R2, Mazhitova and Kaverin 2007), is located in the European part of Russia, on the western side of Ural Mountains (Table S2). Nadym and Vorkuta are both located in the area of discontinuous permafrost, while Vaskiny Dachi is in the area of continuous permafrost.

### MAAT and Snow Depth

In order to estimate the influence of meteorological factors on permafrost dynamics, we used measurements from nine meteorological stations operated by Roshydromet (all connected to World Meteorological Organization network) in Yamal-Nenets Autonomous District (Fig. 1, Table S3). Time series cover period of 2006–2018. The data were downloaded from the site www.rp5.ru (last access February 7, 2019) and were quality controlled (SI). The variables used for the analysis included the air temperature at two meters height, precipitation and snow depth. Using meteorological data, we calculated the mean annual air temperature (MAAT) and mean snow depth during freezing seasons for different years. The MAAT was calculated as the sum of daily mean temperatures divided by the number of days in a year. The mean snow depth for a cold season was calculated as the sum of snow depths, measured on the days when they exceeded zero, divided by the total number of these days.

### Freezing and Thawing Indices

Freezing and thawing indices (or degree-day sums), *I*_f_ and *I*_t_ respectively, were calculated using the following formulas:

1 $$ I_{{\text{f}}} = - \mathop \sum \limits_{{\overline{T} < 0}} \overline{T},\;I_{{\text{t}}} = \mathop \sum \limits_{{\overline{T} > 0}} \overline{T} $$

where the sum is taken over all days during the freezing/thawing season, $\overline{T}$ is the daily mean temperature. The freezing and thawing indices calculated from the air temperature, characterize the annual heat balance indicative of air being cooled or heated during the year. In what follows, we reserve notations *I*_*f*_ and *I*_*t*_ for the freezing and thawing indices calculated based on air temperature. The mean annual air temperature can be calculated as MAAT = (*I*_t _− *I*_f_)/*P*, where *P* is the number of days in the year. Air freezing and thawing indices were calculated directly from air temperature using formula (1). Surface freezing (*I*_f surf_) and thawing (*I*_t surf_) indices can be calculated from Eq. (1) if surface temperature is used instead of air temperature. However, surface temperature is not measured routinely at meteorological stations.

### *n*-Factors

The surface freezing and thawing indices can be calculated multiplying the air indices with the corresponding *n*-factors. Freezing (*n*_f_) and thawing (*n*_t_) *n*-factors, defined as *n*_t(f)_ = *I*_t(f)__surf_/*I*_t(f),_ are the bulk coefficients characterizing heat transfer from air to soil surface on the seasonal time scale and accounting for snow and soil properties in winter and vegetation and soil effects in summer (Klene et al. 2001). We used *n*_t_ = 0.8 as a thawing *n*-factor based on the measurements from boreholes in tundra near Nadym (Kukkonen et al. 2020), see also Jorgensen and Kreig (1988). The freezing *n*-factor *n*_f_ exhibits a greater variability (Kukkonen et al. 2020) and needs to be quantified separately for different years. We derived *n*_f_ as a function of the mean snow depth and MAAT using model calculations by Smith and Riseborough (2002). Similar method was used in a recent study mapping permafrost boundaries (Obu et al. 2019).

We calculated time series of freezing *n*-factors for three meteorological stations: Novy Port, Antipayuta and Vorkuta (Fig. 3a, S2a, S2b). Note that snow depth was not always measured at the station Novy Port which is the closest to the area of anthrax outbreak. For the years when measurements were not available (Table S3, Fig. S1b), we used the mean snow depth calculated from the data of three closest meteorological stations: Yangi-Yugan, Nyda and Nadym (Fig. 1).

### Frost Number

Frost number *F* (Nelson and Outcalt 1987) is a combination of freezing and thawing indices, $F = \frac{{\sqrt {I_{{\text{f}}} } }}{{\sqrt {I_{{\text{f}}} } + \sqrt {I_{{\text{t}}} } }}$, and therefore, accounts for temperature regime during both freezing and thawing seasons. As a reference, the surface frost number of 0.67 was used to mark the boundary between continuous and discontinuous permafrost zones (Nelson and Outcalt 1987). Air and surface frost numbers were calculated from air and surface freezing and thawing indices, respectively. The locations of CALM sites do not coincide with locations of meteorological stations; therefore, to calculate frost numbers, we used the data from closest stations (Nadym for Nadym, Antipayuta for Vaskiny Dachi).

### Summer Precipitation

As a factor influencing spread of the disease, we calculated monthly precipitation as sums of daily precipitation in summer and compared their mean values over periods 2005–2013 and 2014–2018 to the climatological normal of monthly precipitation for 1981–2010. The normals were taken from the Climate Assessment Database (CADBv2) provided by NOAA (last access 15 July 2021).

## Results

### Dynamics of ALT Near Outbreak Location

**Figure 2:**
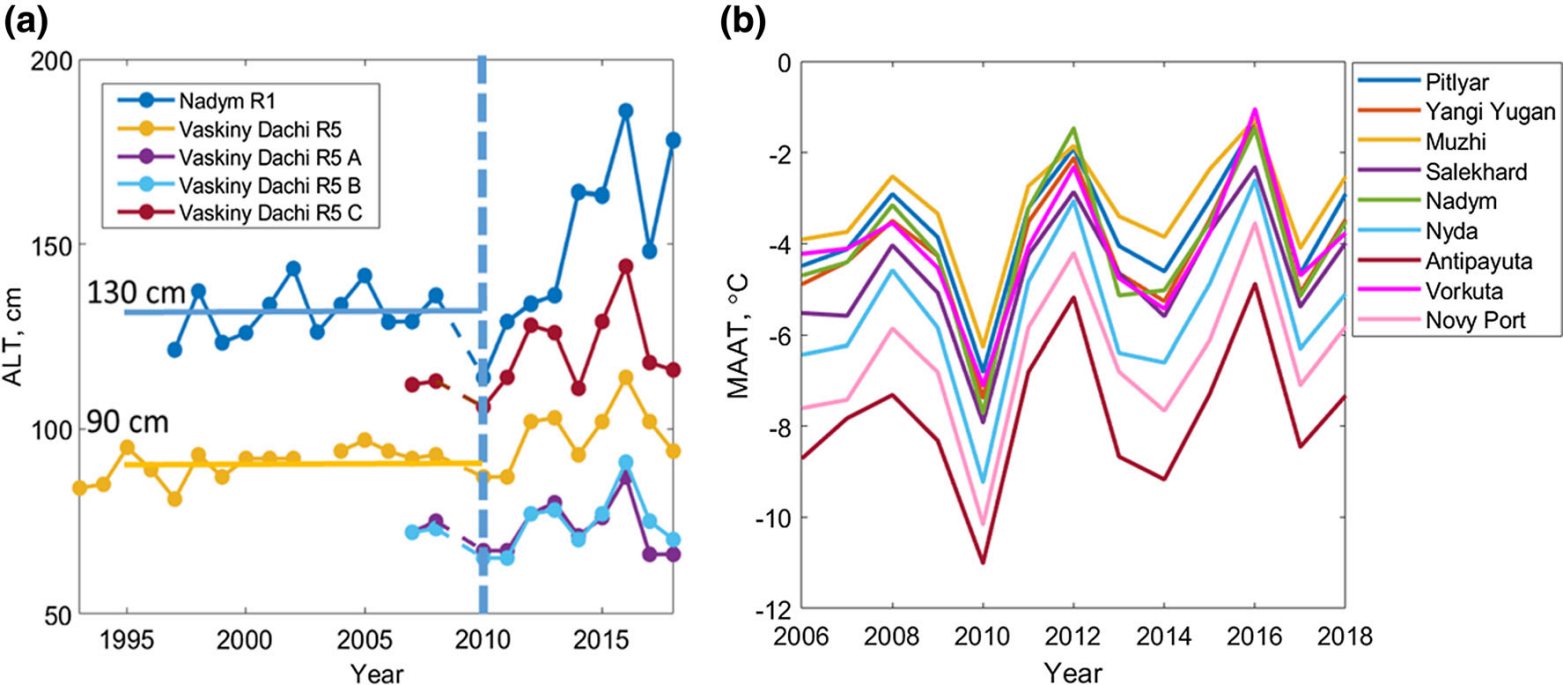
**a** Time series of active layer thickness from Circumpolar Active Layer Monitoring sites. Relative increase in active layer thickness in 2016 as compared to its mean value before 2010 is 43% in the area of discontinuous permafrost (Nadym) and 26% in the area of continuous permafrost (Vaskiny Dachi). Measurements from 2009 are marked as unreliable by the data PIs, therefore dashed line is used between 2008 and 2010. **b** Mean annual air temperature for all sites. Note similar dynamics of active layer thickness in Vaskiny Dachi and mean annual air temperature.

The geographical location of the site of a major reindeer epizootic and human infection cases (Popova et al. 2016) is shown in Figure 1a (see also Supplementary Information, Table S1). All these cases occurred close to the boundary separating zones of continuous and discontinuous permafrost (Kotlyakov and Khromova 2002; Obu et al. 2019). We analyzed the behavior of ALT from three Circumpolar Active Layer Monitoring (CALM) sites closest (200–400 km) to the outbreak location (Novy Port, Fig. 1a): one to the north (Vaskiny Dachi, continuous permafrost), one to the south (Nadym, discontinuous permafrost) and one to the west (Vorkuta, discontinuous permafrost). In contrast to the heat wave hypothesis, the rate of thawing was not enhanced in any of the sites in 2016 (Fig. 2a, Fig. S1c). The maximum values of ALT in Vaskiny Dachi and Nadym followed the trends already started earlier: according to Pettitt’s test, the change point is 2011, *p* value is 0.02. ALT was deepening continuously in Nadym from the minimum in 2010 up to 2016, reaching more than 40% higher level compared to the average value of ALT during 1997–2010. In Vaskiny Dachi, the continuous thawing was interrupted by freezing in 2014, so that the ALT increase was somewhat smaller. The dynamics of the active layer in Vaskiny Dachi show general agreement with the behavior of the mean annual air temperature (MAAT, see Methods) (Fig. 2b), as expected for the cold sites underlain by continuous permafrost (Smith and Riseborough 2002). However, the dynamics of ALT in Nadym cannot be explained by warming alone.

Another important factor for permafrost thaw in the discontinuous permafrost area is the snow depth (Williams and Smith 1989; Stieglitz et al. 2003). Acting as an insulator, snow prevents heat transfer between the cold air and soil surface, thus suppressing freezing of soil in winter. We identified a regional pattern in the snow depth near the Gulf of Ob (Fig. 1c), having roughly 50% higher values in 2014–2015 compared with the period 2006–2013. The average snow depth and the maximum snow depth reached 85 cm (Fig. S1b) and 160 cm, respectively, at one of the stations in 2014. The outbreak of anthrax occurred in the area of deep snow, close to the Gulf of Ob (Fig. 1). Contrary to this, there was no increase in the snow depth during 2014–2015 in the sites closer to Ural Mountains, its mean value fluctuating around 40 cm (Fig. 1b). A snow depth should not exceed 50 cm for permafrost occurrence in tundra near Vorkuta (Mazhitova and Kaverin, 2007; Shamanova 1970). The anthrax outbreak occurred at higher latitudes, near Novy Port, so the corresponding critical snow depth for permafrost occurrence is higher (Smith and Riseborough 2002). However, the drastic snow cover increase of 2014–2015 exceeded the critical values for permafrost occurrence even for the lower MAAT of − 6..− 7°C observed at Novy Port. While this short-term increase in the snow depth did not lead immediately to permafrost degradation, such deep snow certainly kept the soil warm during several winters in a row. More heat energy of the following thawing seasons could be expended directly to thaw permafrost, rather than to warm deeply frozen soil after the cold winter.

### Joint Effect of Snow and Temperature on Permafrost Thawing

**Figure 3:**
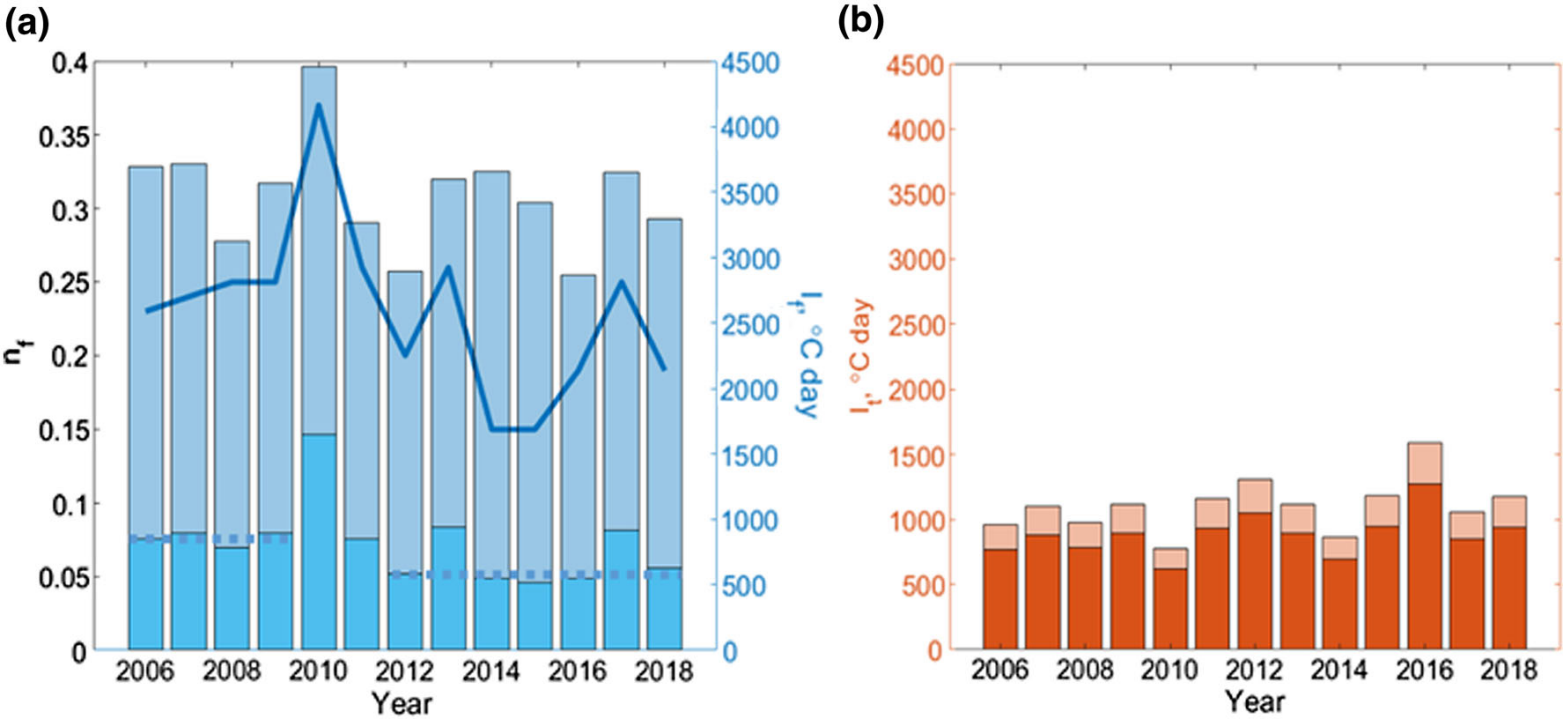
**a** Air freezing index (light blue bars), surface freezing index (cyan bars) and freezing *n*-factor (blue curve) at Novy Port. Straight dashed lines indicate mean surface freezing index in 2006–2009 and decreased index in 2012, 2014–2016. **b** Air thawing index (light red bars) and surface thawing index (red bars).

Freezing indices for surface and air, together with estimated *n*-factors for Novy Port, are shown in Figure 3a. The difference between the air and surface freezing indices is remarkable. The air freezing index was characterized by small-amplitude oscillations near the constant mean value, 3400°C day, with an exception of a maximum in 2010. The soil surface freezing index was relatively constant before 2010, after which it was lower by approximately one third in 5 years out of eight, due to either warm winters or thick snow. The air freezing index was equal or higher than its mean value after 2010 during 6 years out of 8, whereas the surface index was lower than its mean value after 2010 in 5 years out of 8. In 2012 and 2016 when the snow depth was close to 40–50 cm (Fig. 1c, S1b), the *n*-factor was relatively high but the air index was low (winters were warm). Oppositely, in 2014–2015, the air index was high, but the *n*-factor was low due to deep snow (Fig. 1c, S1b). These two factors, warm winters and deep snow, caused persistently lower surface freezing index. These dynamics of the surface freezing index are in qualitative agreement with the ALT dynamics in Nadym. A rapid deepening of the active layer was observed in 2014 (Fig. 2), characterized by the largest snow depth in this region. Thawing indices (Fig. 3b) also increased by ca. 15% as compared to the mean value before 2010, indicating warmer summers.

In Antipayuta, the most northern meteorological station considered here and closest to Vaskiny Dachi, the soil surface freezing index after 2010 was characterized by strong oscillations with large amplitudes (Fig. S2a). However, the lowest surface freezing indices were presumably associated with warm winters rather than with deep snow. Strong MAAT oscillations lead to destabilization of permafrost state, especially if warmer winters are followed by warm summers. Oppositely, for Vorkuta, we found that the soil surface freezing index oscillated near the constant mean value, 730 °C day, during the whole period of observations (Fig. S2b). Note the high freezing *n*-factor in 2016 due to the thin snow cover (Fig. S1b), causing a deep freezing of soil in winter and moderating the effect of the heat wave on permafrost. However, ALT in Vorkuta is generally steady (Fig. S1c), suggesting specific soil properties, most probably characterized by an ice-enriched upper permafrost layer (Mazhitova and Kaverin 2007). In this case, the ‘zero curtain’ (phase transition) periods can be long, leading to a lower sensitivity of permafrost to ambient conditions. Another explanation for a steady ALT in Vorkuta is soil subsidence due to thermokarst (Mazhitova and Kaverin 2007).

**Figure 4:**
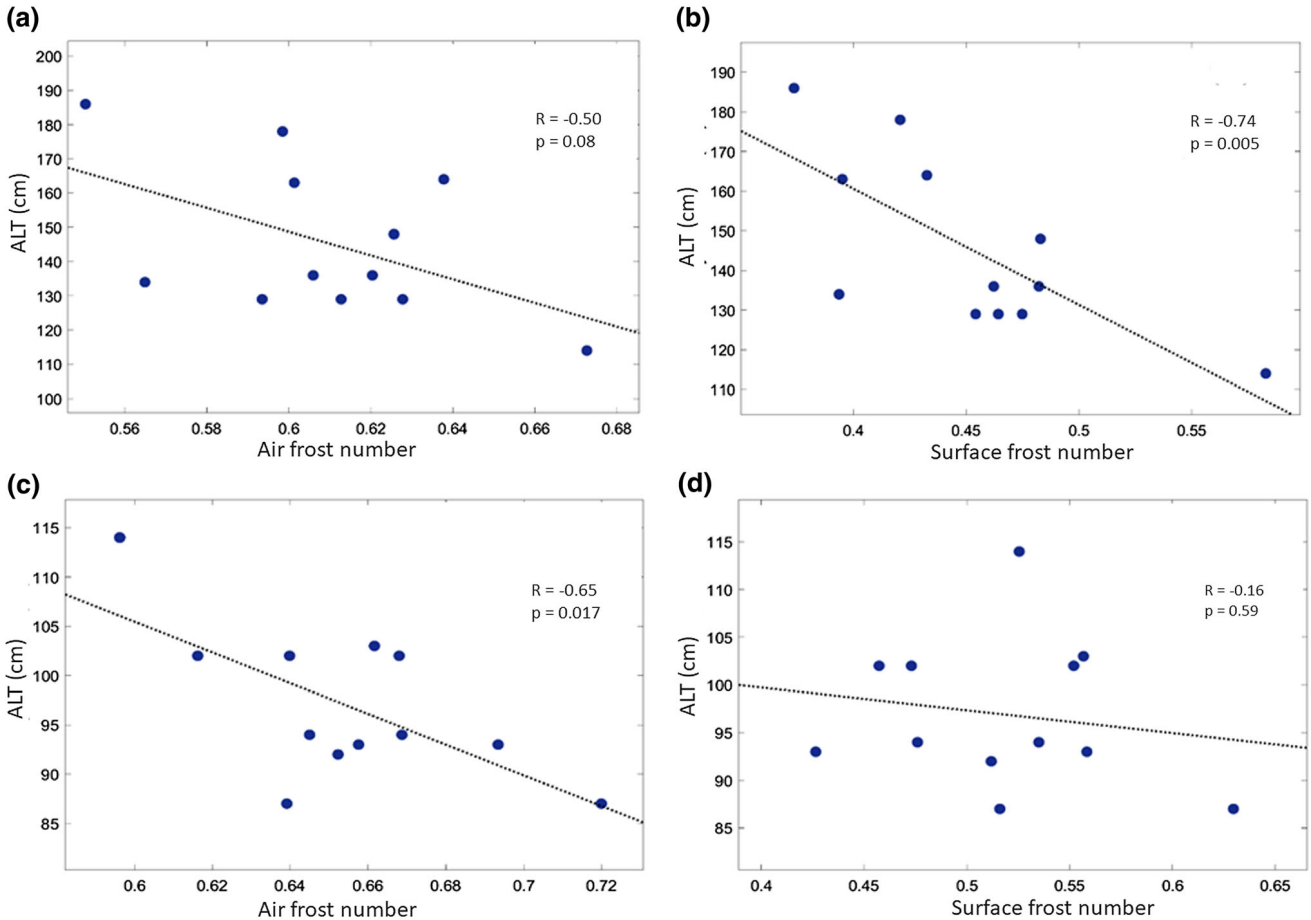
Correlations of active layer thickness with air and surface frost numbers at Nadym (R1) (**a**, **b**) and Vaskiny Dachi (R5) (**c**, **d**).

The combination of freezing and thawing indices, a frost number (Methods), was used for linking meteorological parameters influencing permafrost dynamics and ALT. From Figure 4, ALT in Nadym was significantly and strongly correlated with the surface frost number (Pearson *R* =  − 0.73, *p* = 0.005) rather than the air frost number, indicating that snow was an important factor. Oppositely, ALT in Vaskiny Dachi was significantly and moderately correlated with the air frost number (Pearson *R* =  − 0.65, *p* = 0.017), suggesting air temperature as the major driving factor of permafrost dynamics (see also Fig. 2). The latter could be a consequence of the snow distribution characteristic of Central Yamal (Vaskiny Dachi), which is highly uneven due to low tundra vegetation and strong winds (Leibman et al. 2015). The region of outbreak was located between Vaskiny Dachi and Nadym. The dynamics of permafrost there could carry features of either site, but the most effective thaw could be naturally expected at the sites accumulating snow. The increase in ALT between 2010 and 2016 due to the temperature effect alone was 26% (estimate for Vaskiny Dachi), but deep snow enhanced this effect resulting in 43% increase in ALT (estimate for Nadym). Additional characterization of permafrost thaw using temperature on the top of permafrost for three sites is given in SI (Sec. S2, Fig. S5).

### Summer Precipitation

**Figure 5:**
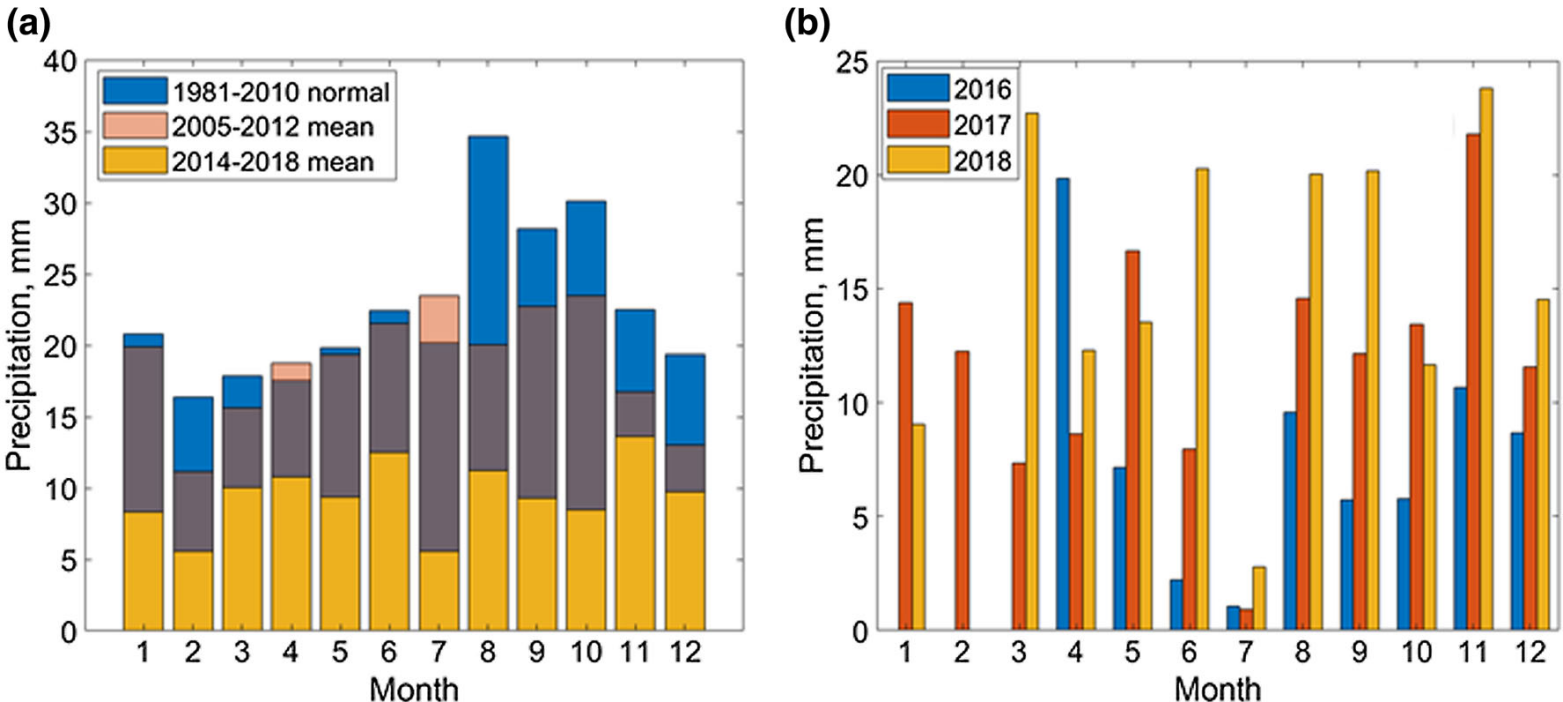
**a** Month-to-month variability of precipitation and NOAA climatological normal of precipitation for 1981–2010 in Novy Port. Note that for 2005–2012, data quality is not sufficient (i.e., up to 50% of measurements per month could be missing), nevertheless the monthly precipitation values in summer exceed those from 2014 to 2018. Data quality for 2014–2018 is acceptable (i.e., less than 15% of data for each month was missing, Table S4). Note decrease in summer precipitation during years 2014–2018 in comparison with climatological normal (see also Fig. S3). Dynamics of precipitation is in accordance with the Second Roshydromet Assessment Report on Climate Change in the Russian Federation, which identifies this region as one with the strongest decreasing trends in annual precipitation (100 mm during 1936–2010). **b** Monthly precipitation in 2016–2018.

Oppositely to snow, the summer precipitation in Novy Port decreased during the latest years (Fig. S3). A decreasing trend in precipitation was previously observed at the station Mys Kamenny (Frey and Smith 2003), 70 km to the north from Novy Port. Note that summer precipitation at the three stations within the distance of only 200 km from each other (Nadym, Nyda and Novy Port, Fig. 1) showed different dynamics during the recent years (Fig. S3). Before 2005, precipitation dynamics in Nyda and Novy Port were similar, suggesting the same mechanisms governing precipitation, and at both sites precipitation decreased. However, after 2005, precipitation patterns were completely different with a rise in Nyda compared to a substantial decline in Novy Port. One explanation for this difference could be a change in vegetation in response to warming near Nyda where a greening trend was reported during 2000–2014 (Miles and Esau 2016). In Novy Port, monthly precipitation in July 2016 was only 5% of the climatological normal for 1981–2010 (Fig. 5), and in Antipayuta, it was below 20% (Fig. S4). When drought occurred, plants extracted water from deeper soils which could bring anthrax spores to the surface (Hugh-Jones and Blackburn 2009). In addition, the lack of precipitation increased the probability of reindeer infection through chewing dry grass and contributed to the high transmissivity of the disease by blood-sucking insects such as tabanids, known to be more active in warm and dry weather (Gainer 2016).

## Discussion and Conclusion

### How Climatic Factors Contribute to the Anthrax Outbreak: An Outline

**Figure 6:**
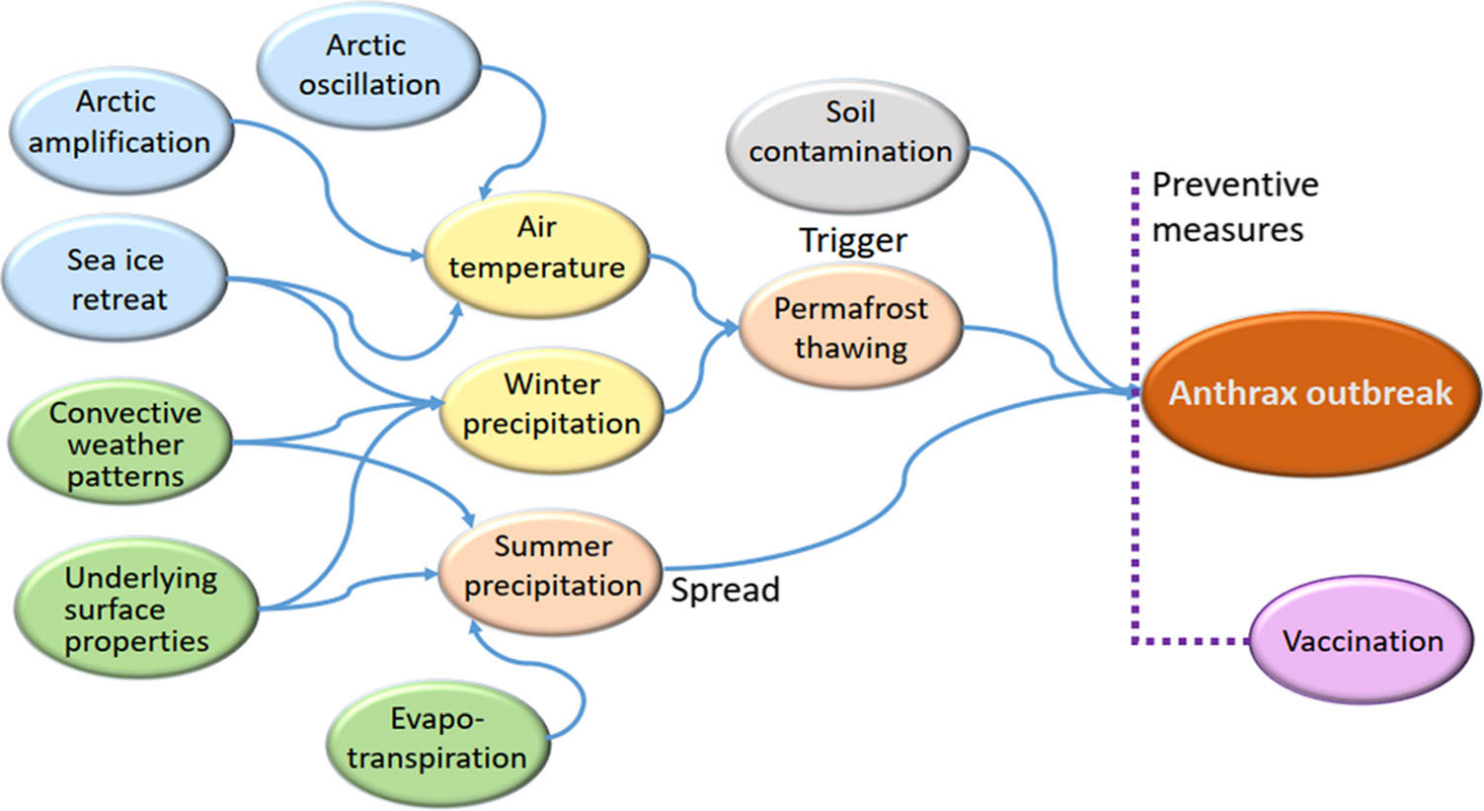
Outline of the connections between climatic factors and anthrax outbreak in the Arctic. Arctic amplification, Arctic oscillation and sea ice retreat determine temperature dynamics in the Arctic on annual scale. Sea ice retreat introduces an increasing trend into the winter precipitation dynamics, whereas local weather patterns and extreme events contribute to its variability. Summer precipitation is determined by the local underlying surface properties, evapotranspiration and convective patterns. Warming climate and winter precipitation dynamics influence active layer deepening which can trigger anthrax outbreak via revival of old bacteria. Dry summer boosts spread of the disease and intensifies the outbreak. Vaccination is a preventive measure to control the spread of disease.

Overall, the schematic of how different factors could contribute to anthrax outbreak in cold climates is shown in Figure 6. For a fixed population of livestock (reindeer), the availability of spores in soil (previous contamination) is of utmost importance, whereas timing, dispersion and incidence rates are connected to climatic factors. Some factors drive permafrost thaw, thereby acting as a trigger of outbreak (warming, deep snow), while others contribute to the spread of disease (lack of summer precipitation).

The increasing trend in the mean annual temperature (Frey and Smith 2003) reflects enhanced warming of Arctic as compared to lower latitudes (Arctic amplification, Box et al. 2019). Fluctuations in the mean annual temperature, showing a similar behavior at all the stations (Fig. S1), are largely driven by the dynamics of the Arctic oscillation (Frey and Smith 2003) which determines winter-time synoptic activity in the region. Arctic oscillation is subject to a random variability and has been mainly in its positive phase since 1990s (NOAA, Climate Indicators), which has led to warmer winters in northern Eurasia, including West Siberia.

Another influencing factor is precipitation (Waits et al. 2018). There has been a general smooth increase in winter precipitation related to sea ice retreat (Cohen et al. 2014). However, the rapid permafrost thaw described here happened due to an anomalously deep snow, pointing at extreme weather contribution, also mentioned by Hedlund et al. (2014) as the factor increasing risk of infectious diseases in Arctic and subarctic regions. In agreement with the previous study showing a high spatial variability in annual precipitation (Frey and Smith 2003), we found a high variability in the snow depth at different meteorological stations over the spatial scale of ca 300 km (Fig. 1). Summer precipitation is dependent on local circulation patterns and properties of the underlying surface. As compared to winter precipitation, it varies on the scale of only 100 km (Fig. 5, S3).

Finally, a major anthropogenic factor influencing the probability of outbreak is vaccination. Vaccination of reindeer had long been a successful preventive measure in this region (Popova et al. 2016; Kolonin 1969). Its role is to prevent occasional infection and to stop a spreading of the disease (WHO 2008).

### Results in the Context of Literature and Implications for Future Research

Climate change introduces a risk of the global anthrax outbreak, both in lower and higher latitudes (Kangbai and Momoh 2017). A recent study mapping global distribution of *B. Anthracis* (Carlson et al. 2019) admits lack of the data on the outbreaks in northern latitudes. Here, we provide the summary of the data characterizing the anthrax outbreak of 2016 in Siberia, and we present the analysis of climatic factors leading to the outbreak. Notably, it was suggested (Carlson et al. 2019) that a set of climatic factors causing outbreaks in the cold climates could differ from that in the warm and dry ones. Based on the present case study, we identified winter precipitation as an additional factor, which has not been considered before. However, we admit lack of replicate examples for anthrax outbreaks in the cold regions, which is among the limitations of this study.

Another previous study considering risk factors for outbreak in high latitudes (Hueffer et al. 2020) admitted the importance of vaccination and reindeer number but provided only a limited analysis of climatic factors, focusing on summer temperatures in Salekhard. We performed an extensive analysis of recent temperature and precipitation and linked it to the dynamics of active layer thickness characterizing permafrost thawing.

The major hypothesis about the trigger of outbreak is related to thawing permafrost. There are also questions on how the outbreak has become so widespread. Given that ALT was increasing since 2010, bacteria could be released from permafrost even earlier than in 2016. The ability of bacteria to undergo the whole life cycle in the soil is a subject of debate (Hugh-Jones and Blackburn 2009). Recently, earthworms, plants and amoebae have been shown to interact with *B. anthracis,* demonstrating a possibility of bacteria’s life cycle outside the host, although in laboratory conditions (Carlson et al. 2018). The contaminated areas in Siberia were considered to be prone to sanitation (Popova and Kulichenko 2017) due to unfavorable soil environment and weather conditions that could abrupt the life cycle of bacteria during an eventual vegetative stage. However, the situation has changed. Since 1968 when Cherkassky examined soil probes (Cherkassky 2003), the growing season (the period with mean daily temperature above 5°C) in Novy Port has lengthened by almost a month and the mean temperature of the growing season has increased by 1°C (Sizov et al. 2021). The climate has become less harsh. In addition, the current vegetation trends show active greening in Yamal district (Miles and Ezau 2016; Sizov et al. 2021), which could enrich soil with organics. Milder cooling of soils during two years prior to the outbreak, together with warmer and longer summers, could create conditions for bacteria to complete their life cycle by increasing the amount of spores if those were released from permafrost earlier than in 2016.

Our analysis points at the importance of the local climatic factors for the outbreak. A combination of climate factors acting for several years in a row caused a strong regional effect over a spatial scale of only 100 km. These scales represent a challenge for the studies based on global models (Walsh et al. 2018; Carlson et al. 2019) hindering the use of large-scale models for prognostic purposes. Regional models with higher resolution can therefore be recommended for the monitoring and forecasts of weather conditions causing unfavorable epidemiological situations in cold climates. In addition, mathematical models can be applied on a local scale (Friedman and Yakubu 2013; Saad-Roy et al. 2017; Gomez et al. 2018; Stella et al. 2020).

Remarkably, long-term climate dynamics indicated risks long before the outbreak. In Northwest Siberia, the mean annual air temperature and snow depth increased by 0.4–0.6°C and 4–10 cm per decade, respectively, during 50–60 years before 2012, while annual precipitation decreased by 50–100 mm in 75 years (1936–2010) (Katsov et al. 2014). Thus, in the absence of vaccination, the dramatic consequences could likely be a question of time. The risk of anthrax outbreaks associated with climate change was pointed out for East Siberia by Revich and Podolnaya (2011). A proper information campaign on the importance of vaccination and prognosis on unfavorable meteorological and climatological conditions is essential to prevent outbreaks in future.

## Supplementary Information

Below is the link to the electronic supplementary material.

Supplementary file1 (DOCX 961 KB)
