# Supplementary material for: Climatic Factors Influencing the Anthrax Outbreak of 2016 in Siberia, Russia
Source: Ecohealth. 2021 Aug 28;18(2):217–28. doi: 10.1007/s10393-021-01549-5 (PMC8463397; doi:10.1007/s10393-021-01549-5)
Supplement: Supplementary file 1 — Supplementary file1 (DOCX 961 KB) [file 10393_2021_1549_MOESM1_ESM.docx]

**Supplementary information (SI)**

**S1 Data sets and quality control**

**S1.1 Medical data set**.

Beginning of epizootic: supposedly 7 July 2016, in the herd of the local farmer. Location: lake Pisyeto, Yamal peninsula (Popova et al., 2016).

Localization: All cases except one were registered on Yamal Peninsula (Popova et al., 2016), see map in Fig. 1. One exceptional case in the beginning of August was registered on Gydan Peninsula, near Antipayuta (Fig. 1).

Infected reindeer (*Rangifer tarandus*) – 2650 cases (2350 died, 300 killed to prevent further contamination).

Infected humans – 36 cases (1 casualty)

Table S1. Statistics on a number of infected humans (registered in hospitals).

**S1.2 Active layer thickness**

Table S2. Description of data sets on active layer thickness (ALT) from Circumpolar Active Layer Monitoring sites.

| CALM site | Location | Years | Missing data, ALT |
| --- | --- | --- | --- |
| Nadym, R1 | N 65° 20' E 72° 55' | 1997-2018 | 2009 |
| Vaskiny Dachi, R5 | N 70° 17' E 68° 54' | 1993-2018 | 2003, 2009 |
| Vaskiny Dachi, R5A | N 70˚16′ 31.8″  E 68˚53′ 29.9″ | 2007-2018 | 2009 |
| Vaskiny Dachi, R5B | N 70˚17′ 43.8″  E 68˚53′00.5″ | 2007-2018 | 2009 |
| Vaskiny Dachi, R5C | N 70˚18′ 05.0″  E 68˚50′28.7″ | 2007-2018 | 2009 |
| Ayach-Yakha, R2 | N 67° 35'  E 64° 11' | 1996-2018 | - |

**S1.3 Meteorological data set**

For the data quality of snow thickness, we applied the following procedure. First, we calculated the number of days when the observations were missing. Second, we removed outliers. An outlier is assumed if the snow thickness is more than 20 cm larger or smaller than the data from the preceding and following days. Given the average length of a winter season about 7 months, for the calculations of the mean snow thickness we accepted only those years when the number of valid measured data points during cold season exceeded 190.

MAAT for the particular year has been calculated for a period from September of previous year to August of the current year in order to account for the effect of the winter and summer on permafrost thawing. Air freezing indices for a given year are calculated for a given cold season, i.e. include part of the winter period from the previous year. For example, freezing index for 2006 corresponds to cold season 2005-2006.

Table S3. Meteorological stations and data quality. Temperature is measured every 3 h (7 times per day), snow thickness is measured once a day during winter season (lasts typically ca 7 months in North-West Siberia). AT is air temperature, ST is snow thickness. Valid data on snow thickness includes only measurements when snow was detected, zero values are excluded.

| Station name (WMO ID) | Geographical location | Analyzed period | Missing AT data, days | Valid ST data, days | Missing ST data, days | Number of ST outliers, days |
| --- | --- | --- | --- | --- | --- | --- |
| Pitlyar (23431) | 65°50’N, 65°55’E | 01.02.2005-31.12.2018 | 6 | 2878 | 169 | 13 |
| Yangi Yugan  (23339) | 66°03’N, 68°41’E | 01.02.2005-31.12.2018 | 9 | 2882 | 311 | 8 |
| Muzhi (23426) | 65°24’N, 64°42’E | 01.02.2005-31.12.2018 | 2 | 2813 | 177 | 3 |
| Salekhard (23330) | 66°32’N, 66°40’E | 01.02.2005-31.12.2018 | 2 | 3026 | 120 | 9 |
| Nadym (23445) | 65°32’N, 72°32’E | 01.02.2005-31.12.2018 | 2 | 2742 | 148 | 13 |
| Nyda (23345) | 66°37’N,  72°57’E | 01.02.2005-31.12.2018 | 3 | 3133 | 156 | 11 |
| Antipayuta (23058) | 69°06’N, 76°52’E | 01.02.2005-31.12.2018 | 34 | 2551 | 212 | 6 |
| Vorkuta (23226) | 67°29’N, 64°01’E | 01.02.2005-31.12.2018 | 2 | 2917 | 108 | 7 |
| Novy Port (23242) | 67°41’N, 72°52’E | 24.06.2005-31.12.2018 | 38 | 1301 | ~1600 | 2 |

Table S4. Meteorological stations and data quality, precipitation. Precipitation is measured every 12 h. In Novy Port, the data from 2013 is missing. In the data analysis, we accounted only for months when >50% of days with good data were available.

| Station name (WMO ID) | Geographical location | Analyzed periods | Precipitation, missing data, days/all days | Summer precipitation, missing data, days/all days |
| --- | --- | --- | --- | --- |
| Antipayuta (23058) | 69°06’N, 76°52’E | 01.02.2005-31.12.2013 | 1888/3254 | 424/736 |
| Antipayuta (23058) | 69°06’N, 76°52’E | 01.01.2014-31.12.2018 | 68/1826 | 38/460 |
| Novy Port (23242) | 67°41’N, 72°52’E | 01.07.2005-31.12.2012 | 1213/2741 | 374/706 |
| Novy Port (23242) | 67°41’N, 72°52’E | 01.01.2014-31.12.2018 | 435/1826 | 61/460 |

**S2 Temperature on the top of permafrost (TTOP)**

Permafrost state can additionally be characterized by the dynamics of mean annual temperature at the top of permafrost (or at the base of active layer), TTOP (Kudryavtsev, 1981; Romanovsky and Osterkamp, 1995). TTOP is calculated using measured surface and subsurface parameters as follows (Smith and Riseborough, 2002)

TTOP = (*r_k_ n_t_ I_t_* – *n_f_ I_f_*)/P, (S1)

where *I_t_* is the air thawing index, *I_f_* is the air freezing index, *n_t_* is the thawing *n*-factor, *n_f_* is the freezing *n*-factor, *r_k_* is the thermal conductivity ratio (the ratio of ground conductivity during thawing season to ground conductivity during freezing season), P is the number of days in the year. Eq. (S1) was derived by Romanovsky and Osterkamp (1995). Increasing TTOP corresponds to thawing permafrost and increasing ALT whereas non-negative TTOP indicates the start of permafrost degradation. Near-surface permafrost exists in the areas where TTOP < 0. When TTOP increases, permafrost thaws.

Below we give a physical interpretation of eq. (1). The surface freezing and thawing indices can be calculated multiplying the air indices with the corresponding *n*-factors. Freezing and thawing *n*-factors, defined as *n_t,f_ = I_t,f surf_/ I_t,f_* are bulk coefficients characterizing heat transfer from air to soil surface on the seasonal time scale and accounting for snow and soil properties in winter and vegetation and soil effects in summer (Klene et al., 2001). From the definition of *n*-factors, the mean annual ground surface temperature (MAGST) can be calculated as MAGST= (*n_t_ I_t_* – *n_f_ I_f_*)/P. Furthermore, conductive heat transfer inside the ground, linking surface conditions to permafrost, differs in summer and winter because of the seasonal differences in the thermal conductivity of soil (in summer lower than in winter). To account for this effect, known as thermal offset, the thermal-conductivity ratio was added in eq. (1) for TTOP into the balance of surface thawing and freezing indices.

Using calculated indices and *n*-factors (Fig. 3, S3a, S3b), we calculated TTOP for mineral soil^21^ with the thermal conductivity ratio *r_k_* = 0.6. Organic soil has a thermal conductivity ratio ranging from 0.3 to almost 1 depending on the soil water content, while in bedrock this ratio is 1^21^.

TTOP is shown in Fig. S5 together with the MAAT time series. Clearly, the oscillations in MAAT contributed to those in TTOP. However, in Vorkuta, the temperature trend in TTOP was absent and oscillations occurred near the constant mean value. This value is close to zero for the mineral soil, but can be negative for organic soils with lower thermal conductivity ratio. In accordance with this result, there was no growing trend in ALT in Vorkuta (Fig. S1c). At the same time, after 2010, TTOP in Antipayuta and Novy Port revealed a transition towards higher mean values. Specifically in Novy Port, mineral soils showed a transition towards non-permafrost conditions (zero mean value of TTOP). In Antipayuta, TTOP revealed strong oscillations indicative of unstable permafrost conditions, in accordance with our index analysis (Fig. S3b).

**SI References**

Kudryavtsev, V. A. (ed.) (1981). Permafrost (short edition) (in Russ.). MSU Press (240 pp.).

Romanovsky V.E. and Osterkamp T.E. Interannual Variations of the Thermal Regime of the Active Layer and Near-Surface Permafrost in Northern Alaska/ Permafrost and Periglacial Processes, Vol 6: 313-335 (1995)

**Supplementary figures**

- (b)

(c)

Fig. S1. (a) Mean annual air temperature at 9 WMO stations. Note cold year of 2010 associated with a strong negative phase of Arctic oscillation. (b) Snow depth at 9 WMO stations. Snow thickness is shown for years when measurements were longer than 7 months. (c) Active layer thickness at three CALM sites.

Fig. S2a. Left panel: Air freezing index (light blue bars), surface freezing index (cyan bars) and freezing n-factor (blue curve) at Antipayuta. Right panel: Air thawing index (light red bars) and surface thawing index (red).

Fig. S2b. Same as Fig. S2a but for Vorkuta.

Fig. S3. Summer (JJA) precipitation in Nadym, Nyda and Novy Port (data downloaded from <http://meteo.ru/data>, last access 15.07.2019). Thick curves show 10-yr running average.

Fig. S4. Left panel: Month-to-month variability of precipitation in Antipayuta and NOAA climatological normal of precipitation for 1981-2010. Gray color appears from transparent red color coinciding with blue. Right panel: Monthly precipitation in Antipayuta, 2016-2018.

- (b)

Fig. S5. MAAT for all sites (a) and TTOP (b) calculated for thermal conductivity ratio 0.6, corresponding to mineral and organic soils. Year corresponds to the hydrological year, from 1 October of previous year to 30 September of the current year. TTOP at Vorkuta oscillates near the constant mean value, while TTOP at Novy Port and Antipayuta are characterized by a shift towards warmer mean temperatures after 2010. Note snow cover effect in 2014-2015 at Novy Port and Antipayuta leading to the shift of TTOP minimum from 2014 to 2013 as compared to MAAT with minimum in 2014. TTOP at Antipayuta, the most northern site in the analysis, shows strong oscillations and depends mostly on MAAT.

Fig. S6. Same as Fig. 4 but for Vorkuta. No significant correlations obtained. The ground subsides in Vorkuta: top of permafrost deepens, but also ground settles (thermokarst), so ALT remains more or less the same. Together with soil properties, this might be another reason for the absence of trends in ALT and absence of correlation between ALT and surface/air frost numbers.
